# Job Demand-Control-Support Latent Profiles and Their Relationships with Interpersonal Stressors, Job Burnout, and Intrinsic Work Motivation

**DOI:** 10.3390/ijerph17249430

**Published:** 2020-12-16

**Authors:** Igor Portoghese, Maura Galletta, Michael P. Leiter, Gabriele Finco, Ernesto d’Aloja, Marcello Campagna

**Affiliations:** 1 Department of Medical Sciences and Public Health, University of Cagliari, 09124 Cagliari, Italy; igor.portoghese@gmail.com (I.P.); gabriele.finco@gmail.com (G.F.); ernestodaloja@gmail.com (E.d.); mam.campagna@gmail.com (M.C.); 2 School of Psychology, Deakin University, Geelong 3220, Australia; leiter.cord@gmail.com

**Keywords:** job demands, job control, job support, latent profile analysis, burnout, social stressors, intrinsic motivation

## Abstract

In the Job Demand-Control-Support (JDCS) model, the combination of job demands, job control, and social support was hypothesized to lead to eight different constellations of job types. According to the model, these constellations are linked to wellbeing/health and learning outcomes. In the last three decades, these constellations of job types have been investigated by adopting a variable-centered perspective. However, latent profile analyses (LPA) enable a person-centered approach and empirically capture constellations of job types. In the present study, we used LPA to empirically identify distinct profiles of JDCS among Italian healthcare workers. Furthermore, we investigated the role of social stressors (workplace relationships and coworkers’ incivility) as antecedents of these profiles and the association of these profiles with job burnout and work motivation. Results from LPA (*n* = 1671) revealed four profiles: Isolated Prisoner, Participatory Leader, Moderate Strain, and Low Strain. Negative relationships at work and coworkers’ incivility increased the chances of being included in both Isolated prisoner and Participatory Leader profiles. Finally, the Isolated Prisoner and Moderate Strain profiles showed the highest levels of emotional exhaustion and cynicism and the lower levels of intrinsic work motivation. This study extends previous JDCS research, highlighting that researchers should consider empirically identified profiles rather than theoretically defined subgroups. Implications for stress theory, future research, and practice are discussed.

## 1. Introduction

**Figure 1:**
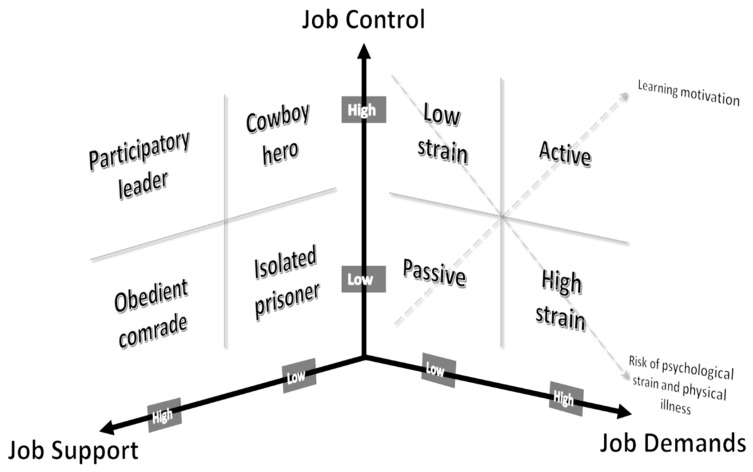
The three-dimensional Job Demand-Control-Support (JDCS) model of psychosocial work environment (adapted from Karasek and Theorell, 1990, p. 70).

The Job Demand-Control (JDC) model [1] has been highly influential in the occupational stress and health literature for more than 40 years [2]. This model and its second theorization, the Job Demand-Control-Support (JDCS) [3], have been the theoretical foundation for more empirical studies than any other work stress model [4,5,6,7,8,9,10,11,12,13,14]. The central hypothesis of the model is that combinations of job demands, job control, and social support are associated with higher strain and reduced wellbeing. In this sense, Karasek and colleagues proposed that the risk linked to the combined exposure of these three factors was higher than the risk associated with each exposure separately [3]. Furthermore, Karasek and colleagues [3] postulated that combinations of different levels (high or low) of these three factors lead to different constellations of job types that, in turn, may lead to particularly adverse work situations (see Figure 1).

In the last three decades, these constellations of job types were considered as predefined exposure profiles. Almost all JDC(S) model related studies have mainly focused on the combinations of job demands, job control, and social support, investigating relationships by traditional regression/correlation techniques. Thus, in considering these patterns of job types, scholars developed a vast scientific literature that strongly relied on variable-centered methodology. Commonly, researchers considered job types as previously defined on the basis of the JDC model, exploring these constellations by applying, for example, median or mean cut-off values or median splits based on sample data [15]. However, these studies are considered limited, mainly because the possibility that the sample should be assumed as not homogeneous [16] is not taken into account. In this sense, the configuration of constellations of job types as postulated in the JDC(S) models has been rarely confirmed empirically and few studies were able to identify all the hypothesized constellations [2,15]. Furthermore, it is unknown how social support is likely to combine with job demands and job control in order to identify the proposed constellations.

Thus, considering this lack of research in the JDCS domain, a person-centered approach will be necessary if researchers are interested in identifying subpopulations of individuals showing different patterns, as postulated by Karasek and colleagues [3]. In sum, the main purpose of this study was to extend previous research on the JDCS model, contributing to a better theoretical and empirical understanding of the model by adopting a person-centered approach to identify relevant job type profiles. Several key questions in the JDCS literature may be better answered by a person-centered approach. For example, are the constellations (profiles) of job types empirically founded? What antecedents and consequences are associated with different profiles?

### 1.1. The JDCS Model: Theoretical Framework

According to the JDC, the imbalance of job demands and job control (originally labelled as decision latitude) may be strongly predictive of employees’ wellbeing. Job demands refers to “psychological stressors involved in accomplishing the workload” [3] (p. 291). Job control refers to two fundamental components: skill discretion and decision authority. The first one refers to employees’ freedom to use specific job skills at work, whereas the second one refers to employees’ autonomy in task-related activities. In extending their model, Karasek and Theorell [3] added social support in the workplace as a third component. Thus, in the new theorization of the JDCS model, social support was defined as “overall levels of helpful social interaction available on the job from both co-workers and supervisors” [3] (p. 69). In this regard, according to the JDCS model, a work environment can be described in terms of the combination of demands that are present in the job, the amount of control that workers have to manage in order to meet those demands, as well as the support they receive from both supervisors and peers.

In its first theorization, the combination of job demands and job control was hypothesized to lead to four different constellations of job types (see Figure 1): high-strain jobs (high job demands and low job control), active jobs (high job demands and high job control), low-strain jobs (low job demands and high job control), and passive jobs (low job demands and low job control). Then, in the JDCS model [3], the combination of job control and social support was hypothesized to lead to four new additional constellations (see Figure 1): participatory leader (high job control and high social support), obedient comrade (low job control and high social support), cowboy hero (high job control and low social support), and isolated prisoner (low job control and low social support).

Karasek and colleagues postulated two main hypotheses concerning the link between these constellations, well-being/health and learning outcomes [3,7]. The first one, the strain hypothesis, suggests that high strain jobs (characterized by high job demands and low job control) predict workers’ ill-being (see Figure 1). More generally, authors suggested that jobs characterized by high job demands are expected to have a negative effect on workers’ health. Furthermore, according to the JDCS model, “iso-strain jobs” (characterized by high job demands, low job control, and low social support) expose workers to the highest risk of poor psychological wellbeing and ill-health [3,8]. As for passive and low-strain jobs, as those jobs imply limited challenges and opportunities from work, the model postulates that these jobs may expose workers to moderate or low levels of wellbeing [1]. Increasing job demands and decreasing job control (and job support) would then underlie this hypothesis, which would be related to an increase in strains and several indicators of ill-health [9]. The second main hypothesis concerns the learning hypothesis [1], suggesting that high job demands together with high job control predict higher levels of work motivation, learning, skill development, and increased personal growth at work. In this sense, active jobs (high job demands and high job control) may promote learning, motivation, and personal growth at work. In the “learning hypothesis”, the assumption is that high job demands and high job control promote both activation and learning. In this sense, Karasek suggested that activation and learning should be considered as “an increase in overall activity and in general problem-solving activity” [1] (p. 288).

Both these hypotheses have been tested by adopting the JDC theorization, particularly the strain hypothesis [9,10,11,12,13,14]. However, no explicit hypotheses were proposed concerning the relationship between participatory leader, obedient comrade, cowboy hero, and isolated prisoner job types and ill-being and learning outcomes. Considering the nature of these jobs, participatory leader jobs are supposed to be linked to higher wellbeing as workers have sufficient job control and social support for coping with job demands. Moderate levels of job demands may promote both activation and learning. Obedient comrade and cowboy hero jobs may expose workers to moderate or low levels of wellbeing because of low levels in job control and/or social support. In this sense, those jobs may promote low activation and learning. Finally, isolated prisoner jobs are in line with the “iso-strain jobs” constellation and then expose workers to the lowest level of wellbeing and learning.

### 1.2. Application of the Person-Centered Approach to the JDCS Model

The empirical identification of subpopulations could extend JDCS theory by either offering new insight into the job stress research and solving ambiguous perspectives and findings on the relationships among job constellations and health outcomes. Therefore, several key questions in the job stress literature may be better answered by a person-centered analytic approach.

In the last three decades, the variable-centered perspective has dominated the job stress field so far [15] and almost all JDC(S) models have employed a variety of different methods to investigate the model. In this sense, scholars strongly relied on variable-centered techniques, such as descriptive statistics, correlations, and multiple regression. Furthermore, when interested in investigating and examining the relationships of job constellations with outcomes, median split techniques were used for identifying such job patterns or “profiles”. In fact, the use median split procedures allow researchers to rank participants as “high” or “low” if their score is above or below the median for the factors considered respectively (in our study, job demands, job control, and job support). Although this technique is easy to implement, the numerous problems inherent in the use of the median are now well known. MacCallum, Zhang, Preacher, and Rucker [16] in their review suggested that one of the main problems related to the use of median split procedures is linked to the questionable homogeneity of the classification of subjects in each profile, as well as the problematic use of labels such as “low” and “high” to classify cases falling below and above the median.

According to Morin, Morizot, Boudrias, and Madore [17], variable-centered approaches “ignore the fact that participants may come from different subpopulations in which the observed relations between variables may differ, quantitatively and qualitatively” (p. 59). Compared with a variable-centered approach, a person-centered approach is able to relax the assumption of homogeneity in the population through the evaluation of a different set of parameters. This approach refers to statistical methods that probabilistically assign individuals into subpopulations [18]. Latent class analysis (LCA) and latent profile analysis (LPA) are special cases of finite mixture models that aim to identify naturally occurring latent subpopulations that are homogeneous in relation to selected criteria variables [18]. In this sense, this approach may help researchers to understand if different latent (unobserved) groups of individuals share several patterns of job demands, job control, and job support.

Few studies adopting a person-centered approach in investigating constellations of jobs as postulated in the JDCS model have been carried out so far. For example, Holman [19] and De Spiegelaere, Ramioul, and Van Gyes [20], through adopting different theoretical frameworks, specifically the Job Demand-Resource Model (JD-R) [21], were able to identify different constellations. Specifically, Holman [19] found three of the four constellations (high strain, active jobs, and passive jobs) postulated by Karasek [1] in a two-step cluster analysis. Similarly, De Spiegelaere and colleagues [20] identified in their study both high- and low-strain jobs as postulated in the JDCS model. Mauno and colleagues [2] adopted a longitudinal person-centered approach to the JDC model and found support for high- and low-strain profiles. However, they considered only two of the three components of the JDCS model, thus ignoring social support from their analyses. Igic, Keller, Elfering, Tschan, Kälin, and Semmer [22], in a longitudinal person-centered approach, found empirical support for three constellations: active jobs, low-strain jobs, and high-strain jobs. Mäkikangas, Tolvanen, Aunola, Feldt, Mauno, and Kinnunen [23] investigated job profiles based on the JDCS model in a multilevel LPA. Their results showed support for only two profiles: collective low-strain jobs and isolated high-strain job. Recently, in their study based on the JD-R model, Lee and Cho [24] combined job demands, job resources (skill discretion, decision authority, co-worker support, and supervisor support), and personal resources (self-esteem, optimism, and active coping), finding different profiles that would not theoretically be linked to the JDCS model. Finally, Gameiro, Chambel, and Carvalho [25] investigated job profiles based on the JDC model (thus ignoring social support), finding support for all the four hypothesized profiles in the JDC model (high-strain, low-strain, passive, and active job types). Furthermore, the authors found one more profile denominated “moderate active”, confirming that in contrast with the theoretically defined subgroups of the JDC model, new and different empirically identified profiles may emerge.

Overall, results from these studies partially identified the constellations proposed by the JDCS model [3], emphasizing the need for considering a change in the analytical perspective as “one would not expect to see the full set of quadrants represented” by the JDCS model [26].

### 1.3. Predictors of Job Stress Profiles

The second aim of our study was to investigate how predictors might affect profile membership. To the best of our knowledge, no research has ever analyzed the role of predictors in shaping JDCS profiles. In variable-centered research, relatively little attention has been focused on the study of the conditions involved in predicting job types, as postulated in the JDCS model, except for high-strain jobs that have been extensively studied [27].

In the last decade, increasing attention has been paid to the role of social stressors, such as negative social interaction at work (e.g., interpersonal mistreatment behaviors) in the stressor-strain process [28,29]. As humans are social beings, the quality of interpersonal relationship they develop would significantly influence their attitudes and behaviors [30]. According to Klumb, Voelkle, and Siegler [31], “negative social interactions at work are characterized by negative form or content with varying degrees of intensity, such as criticism, lying, rude comments, derogatory remarks, yelling, or insults from supervisors or coworkers” (pp. 630–631). Despite the extensive research in this area, no standardized definition of negative relationships has been developed yet. By focusing on the verbal interaction, Morrison [32] proposed that it is a communication-related interaction characterized by “passive to active dislike, animosity, and disrespect” (p. 331). In this sense, negative social interaction should be considered a climate component that defines the social environment where JDCS profiles may develop. While working in this kind of context, workers are required to devote a significant amount of energy in order to deal with negative workplace interactions, which may influence the way workers perceive job demands, job control, and social support.

Furthermore, workplace incivility can be another important source of negative social interaction at work. It has been defined as “low-intensity deviant behavior with ambiguous intent to harm the target, in violation of workplace norms for mutual respect” [33] (p. 457). Uncivil behaviors are characterized by rudeness, discourteousness, and lack of concern for others. Workplace incivility can have multiple sources, including coworkers, superiors, or even clients/patients [34].

Andersson and Pearson [33] stated that because “in every workplace there exist norms for respect for fellow coworkers” (p. 445), coworker incivility refers to the behavior of “acting rudely or discourteously without regard for others, in violation of norms for respect in social interactions” (p. 445). In the traditional variable-centered research, coworker incivility has been found to contribute to experiencing increased levels of job stress [34,35,36,37,38,39]. Oore and colleagues [40] have found that workplace incivility from coworkers would reinforce the relationships between employees’ workload and mental health and job control and mental health.

Consistent with this perspective, our objective is to explore whether profile membership would be predicted by negative relationships at work and coworkers’ incivility.

### 1.4. Outcomes of Profile Group Membership

The third aim of our study was to investigate both strain and learning hypotheses. There is general evidence that job stress is significantly associated with several job and health outcomes. According to the strain hypothesis of the JDCS model [3], high isolation strain jobs, characterized by high job demands, low job control, and low social support, can have an adverse effect on workers’ wellbeing/health. Traditional variable centered research has showed how high job strain is linked to poor mental health [41,42], burnout and more job-related psychological distress [43,44,45], and poor physical health [26,46].

According to the learning hypothesis, low-strain jobs are supposed to not provide stimulating or inspiring involvement at work that are expected to enhance positive work motivation. Little research has focused on the learning hypothesis and recently, a study by Mauno and colleagues [2] has shown mixed results that were only partially supported by the learning hypothesis. More specifically, the study has shown that (stable) low-strain jobs show lower exhaustion (in line with the strain hypothesis) and higher work motivation (not in line with the learning hypothesis) outcomes when compared to high-strain jobs. Results suggested that high job demands are not linked to higher work motivation. In the traditional variable-centered studies, De Spiegelaere and colleagues [20] found support for this hypothesis, showing that active jobs (high job demands and high job control) promoted innovative work behaviors and work engagement.

To our knowledge, limited research has tested both the strain and learning hypothesis in a person-centered perspective. Thus, examining the linkages between JDCS profiles, job burnout, and work motivation might offer new information about these relationships that has not received extensive attention in previous variable-oriented studies.

### 1.5. Research Questions

Our study was guided by the following research questions:

Research Question 1: Are there quantitatively and qualitatively distinct profiles of JDCS?

Research Question 2: Do negative relationships at work and workplace incivility predict profile membership?

Research Question 3: Do JDCS profiles exhibit different levels of job burnout (emotional exhaustion and cynicism) or work motivation?

## 2. Materials and Methods

### 2.1. Ethical Considerations

According to the Italian law 211/2003, no formal ethical approval is required for observational studies in the absence of any involvement of therapeutic medication. By therefore complying to the law, an approval by a Medical Ethical Review Committee is not needed. Nonetheless, all workers received verbal and written information about the purpose of this study. Subsequently, informed consent was obtained after the questionnaire-filling session. Our research complies with the Declaration of Helsinki as well as with Italian privacy law (Decree n. 196/2003). We assured that no details about participants identities would be reported, therefore the findings have just been reported in aggregate form. Participants were informed that their participation in the research was voluntary and that they could cease participation from the study at any time, for any reason, and without adverse effects on their employment. Moreover, we assured that the data would be kept in strictest confidence.

### 2.2. Data Collection and Participants

A cross-sectional survey study has been carried out. Data were collected as a part of a larger organizational procedure for psychosocial risk assessment. During the period of January 2016–July 2017, a self-reported paper questionnaire was administered to 3611 healthcare workers from five Italian hospitals. Survey packets, which included a cover letter explaining the study and a questionnaire, were distributed. Participation in the study was voluntary and participants completed the anonymous questionnaire during working hours. Participants were given 3 weeks to complete and return the completed questionnaire in a closed box in a ward conference room. The time required to fill in the questionnaire was about 15 min.

A total of 2010 questionnaires were returned, yielding a response rate of 55.7%. Following this, 339 questionnaires were removed because of missing responses (>5%) about variables relevant to this study.

The sample (*n* = 1671) was predominantly female (68%). As for participants’ age, 14.7% of respondents were younger than 32 years, 18.9% were aged 33–39, 32.6% were aged 47–55, and 31.6% were older than 55. Concerning job tenure, 15.5% had less than 3 years of job tenure, 29.7% between 4 and 10 years, and finally, 47.8% had more than 10 years. Regarding job role, 22.4% were nurses, 30.6% were physicians, and 37.2% were other health professionals (midwives, physical therapists, therapists, social workers, healthcare assistant, nurse aides, and psychologists).

### 2.3. Measures

The Italian version of the HSE Management Standards Indicator Tool subscales [47,48] were used to assess employees’ levels of job demands (8 items; e.g., “I have to neglect some tasks because I have too much to do”), job control (6 items; e.g., “I can decide when to take a break”), managerial support (5 items; e.g., “I can rely on my line manager to help me out with a work problem”), coworkers’ support (4 items; e.g., “I get help and support I need from colleagues”), and negative relationships at work (4 items; e.g., “Relationships at work are strained”). The items were rated using a 5-point Likert scale, ranging from 1 (strongly disagree) to 5 (strongly agree). McDonald’s ω for each subscale were: job demands = 0.88, job control = 0.82, managerial support = 0.84, coworkers’ support = 0.82, and negative relationships at work = 0.76.

The Straightforward Incivility Scale (SIS; 3 items) was used to assess the frequency of uncivil behaviors from colleagues [49,50]. A sample item was: “My coworkers/supervisor spoke rudely to me”. The items were rated using a 7-point Likert scale ranging from 0 (never) to 6 (daily). McDonald’s ω was 0.89.

The Italian version of the Motivation at Work Scale (MAWS) [51,52] was used to assess intrinsic work motivation. Specifically, we used the five-item subscale of intrinsic work motivation. The items were rated through a 7-point scale from 1 (“does not correspond at all”) to 7 (“corresponds very strongly”). A sample item was “Because I enjoy his work very much”. McDonald’s ω was 0.91.

The emotional exhaustion (4 items) and cynicism (4 items) subscales of the Italian version of the Maslach Burnout Inventory-General Survey [53,54] were also used. Participants used a seven-point Likert scale, ranging from 0 (never) to 6 (every day), to rate the extent to which they experience exhaustion (e.g., “I felt emotionally drained from my work”) and cynicism at work (e.g., “I have become less enthusiastic about my work”). McDonald’s ω were 0.78 and 0.73, respectively.

### 2.4. Data Analysis

Preliminary measurement models were assessed using the weighted least squares mean and variance adjusted (WLSMV) in Mplus 7.0 [55].

A series of confirmatory factor analyses (CFA) and exploratory structural equation modeling (ESEM) were contrasted to evaluate the psychometric properties of the adopted instruments. Specifically, given the high complexity of the measurement models of the constructs studied, we relied on previous work [56,57,58] in conducting separate preliminary measurement models. In the first one, preliminary measurement models were estimated on the scales measuring job demands, job control, and managerial and coworkers’ support. Based on these measurement models, we created factor scores to serve as indicators of LPA [59,60]. In the second measurement model, we estimated psychometric properties of antecedent (negative relationships at work and coworkers’ incivility) and outcomes (intrinsic motivation, emotional exhaustion, and cynicism) measures.

Following Morin and colleagues’ procedure [17,61], we contrasted CFA and ESEM models. Recent studies have shown the advantages of using an ESEM measurement model [62,63,64,65,66,67,68,69]. In fact, while in the CFA model each item is only allowed to load on the a priori-defined factor and cross-loadings are not allowed, in ESEM, all cross-loadings are “targeted” to be as close to zero as possible and the main loadings are freely estimated. In this sense, ESEM can minimize biases in structural parameter estimates [62,63,64,65,66,67,68,69]. ESEM models were specified by using a confirmatory approach by means of target rotation [56], which allows for the pre-specification of target loadings in a confirmatory manner, while cross-loadings are targeted to be as close to zero as possible.

We relied on the following common goodness-of-fit indices [70]: the chi-square (χ2), the comparative fit index (CFI), the Tucker-Lewis Index (TLI), the root mean square error of approximation (RMSEA), and the weighted root mean square residual (WRMR). In order to interpret these fit indices, we considered values >0.90 and >0.95 for the CFI and TLI, respectively, by indicating adequate and excellent fit to the data. Values smaller than 0.08 or 0.06 for the RMSEA are acceptable and excellent model fit, respectively, and acceptable WRMR for values ≤1.00.

### 2.5. Latent Profile Analyses (LPA)

LPA was used to extract profiles according to their levels of job demands, job control, managerial support, and peer support. Factor scores that were saved from the final first-order (CFA or ESEM) models in standardized units (M = 0, SD = 1) were subsequently used as profile indicators [69].

LPA including one to eight latent profiles were estimated by using Mplus 7 robust maximum-likelihood estimator (MLR). In order to avoid converging on a suboptimal local maximum, all LPA were conducted by using 10,000 random sets of start values and 1000 iterations, as well as the 500 best solutions retained for final stage optimization [71,72]. In all LPAs, the means and variances were freely estimated [67,68]. The indicators’ intercepts and residuals were freely estimated in all profiles [73,74].

In order to decide how many profiles should be retained, two general criteria were followed: (1) consistency with the theoretical meaning and conformity of the extracted profiles [68], (2) statistical appropriateness of the extracted solution [69,75]. The following goodness-of-fit indices were considered: the Bayesian information criterion (BIC) and Akaike information criterion (AIC), the Constant AIC (CAIC); the bootstrapped likelihood ratio (BLRT) *p*-value and the Lo-Mendell Rubin adjusted likelihood ratio (LMR-A) were used to indicate whether the current model fits data in a better way than a model, which would be composed of a fewer number of classes (one less class) [68,76]. Non-significant *p* values support the k-1 profile model. Finally, a higher entropy value indicates a larger degree of separation between classes. The standard procedure would state as follows: the model with the largest number of classes, the smallest BIC value, and a significant LMR-A, as well as the intelligibility of the profiles, should be the accepted one [77]. Furthermore, information criteria were graphically presented through “elbow plots”, showing the improvements related with additional profiles [68,73,78]. More specifically, the optimal number of profiles should be inspected at the time when the slope flattens by then considering one more and one less profile.

Subjects were assigned to classes according to their posterior class membership probabilities. Subsequently, we then examined associations between assigned membership and external variables. The inclusion of predictors should not qualitatively change the profiles [68]. More specifically, we regressed the latent profiles on coworkers’ incivility and negative working relationships in a series of multinomial logistic regressions. In order to do so, the R3STEP method in Mplus [79] was used. The R3STEP method was used to investigate whether a predictor is related to a higher probability of a participant belonging to one profile rather than another.

Finally, the DU3STEP\DE3STEP methods in Mplus [79] were used to determine whether the means emotional exhaustion, cynicism, and work motivation were different across the latent profiles.

## 3. Results

### 3.1. Preliminary Analyses and Descriptive Statistics

As a first step, we tested the four-factor measurement model for job demands, job control, managerial support, and coworkers’ support. The CFA did not provide a satisfactory degree of fit to the data according to the CFI = 0.835, TLI = 0.818, the RMSEA = 0.122, and the WRMR = 4.366. By contrast, the ESEM model resulted in a substantial improvement in fit, providing a good fit to the data according to the CFI = 0.956 and TLI = 0.933, as well as an acceptable level of fit to the data according to the RMSEA = 0.076. The WRMR only showed a poor level of fit (1.372). Based on this statistical evidence, we retained the ESEM solution. Except for two items showing low target factor loadings in ESEM (job control 5: λ = 0.280; managerial support 1: λ = 0.251), the four a priori constructs were well defined through high target factor loadings (λ = 0.421 to 0.941; M = 0.722).

Separately, we assessed the five-factor measurement model for antecedents (negative relationships at work and coworkers’ incivility) and outcomes (intrinsic motivation, emotional exhaustion, and cynicism) of latent profiles. The CFA did not provide a satisfactory degree of fit to the data according to the CFI = 0.893, TLI = 0.874, the RMSEA = 0.155, and the WRMR = 4.017. By contrast, the ESEM model resulted in a substantial improvement in fit, providing an excellent fit to the data according to the CFI = 0.981 and TLI = 0.962, as well as a slightly acceptable level of fit to the data according to the RMSEA = 0.085. The WRMR showed a slightly acceptable level of fit (1.010). Based on this statistical evidence, we retained the ESEM solution. Except for one item showing low target factor loadings in ESEM (Emotional exhaustion 3: λ = 0.334), the five a priori constructs were well defined through high target factor loadings (λ = 0.334 to 0.957; M = 0.731).

Table 1 presents means, standard deviations, and correlations between study variables.

### 3.2. Latent Profile Analysis

**Figure 2:**
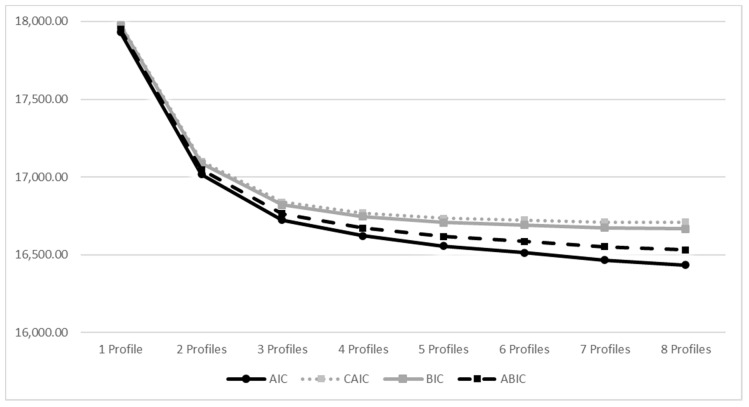
Elbow plot of the information criteria.

Fit indices resulting from the latent profile models containing up to 8 profiles are provided in Table 2. BIC decreased as the number of profiles increased, but the decrease became minimal beginning from the 5-profile to the 8-profile model. However, information criteria have not reached a minimum value and we had to rely on the elbow plot (see Figure 2).

Taken as a whole, the 3-, 4-, and 5-profile solutions showed a better fit as they were supported by the BIC and ABIC values, the aLMR and BLRT tests (Table 2). Nevertheless, the 5-profile solution led to a spurious profile that comprised only 24 individuals (1.45%). According to Hipp and Bauer [71], models with classes representing less than 5% of the sample should not be considered.

Then, we compared the 3- and 4-profile solutions. Comparisons of the AIC, BIC, and SABIC for all the models were contrasted in an elbow plot. Results were based on BIC and SABIC values and the aLMR and BLRT tests also supported the 4-profile solution. The LMR likelihood ratio tests demonstrated significant results when comparing the 4-profile model versus the 3-profile model (*p* < 0.01). Furthermore, this final solution was also examined considering the theoretical meaningfulness of the profiles [67].

**Figure 3:**
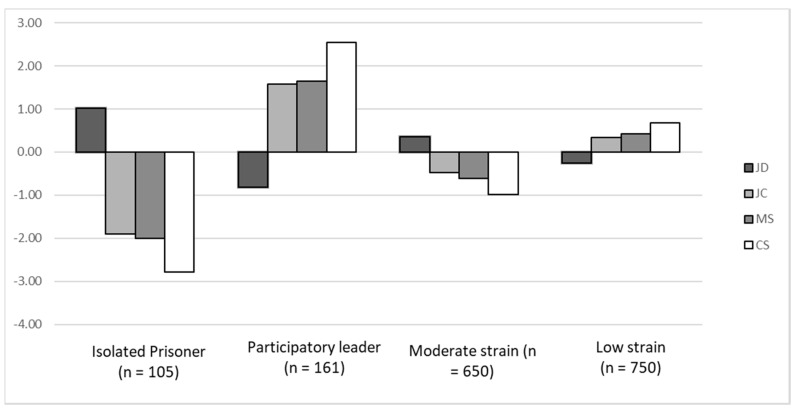
Results from the Latent Profiles Models (standardized values). JD = Job demands; JC = Job Control; MS = Managerial Support; CS = Coworkers’ Support.

Profile 1 represents 6.3% (*n* = 105) of the sample (latent profile membership probability = 0.83) and it is characterized by very high job demands, very low control, very low managerial support, and very low coworkers’ support (Figure 3). Thus, following the JDCS theorization, this profile was labelled as Isolated Prisoner. Profile 2 represents 9.6% (*n* = 161) of the sample (latent profile membership probability = 0.82) and is characterized by low job demands, high job control, high managerial support, and high coworkers’ support. Thus, following the JDCS theorization, this profile was labelled as Participatory Leader. Profile 3 represents 38.9% (*n* = 650) of the sample (latent profile membership probability = 0.81) and is characterized by slightly above average levels of job demands, slightly below average levels of job control, low managerial support, and low peer support. Thus, this profile was labelled as Moderate Strain. Finally, profile 4 represents 45.2% (*n* = 750) of the sample (latent profile membership probability = 0.84) and is characterized by slightly below average levels of job demands, slightly above levels of job control, moderate managerial support, and moderate peer support. Thus, this profile was labelled as Low Strain.

Concerning Research Question 1, these results suggested that quantitatively and qualitatively different job type profiles exist.

### 3.3. Predictors of Profile Membership

Predictors were added to this final 4-profile model. The results from this multinomial logistic regression are reported in Table 3. Results indicated that employees that perceived high negative relationships at work were more likely to be included in the Isolated prisoner profile than the Participatory Leader profile (OR = 99.48, *p* < 0.01), the Moderate strain profile (OR = 5.05, *p* < 0.01), and the Low strain profile (OR = 27.47, *p* < 0.01). Furthermore, high negative relationships at work increased the chances of being included in the Participatory Leader profile when compared with Low strain profile (OR = 3.63, *p* < 0.01). Moreover, high negative relationships at work would increase the chances of being included in Moderate strain profile when compared with Participatory Leader profile (OR = 19.65, *p* < 0.01) and Low strain profile (OR = 5.42, *p* < 0.01).

As for the second predictor, results indicated that coworkers’ incivility would predict the likelihood of belonging to the Isolated Prisoner profile, rather than the Participatory Leader profile (OR = 5.18, *p* < 0.01), the Moderate strain profile (OR = 1.85, *p* < 0.01), and the Low strain profile (OR = 3.56, *p* < 0.01). Furthermore, coworker incivility has shown not to increase the likelihood of being included in the Participatory Leader profile when compared with Low strain profile (OR = 1.46, *p* > 0.05). Finally, coworkers’ incivility predicted the likelihood of belonging to the Moderate strain profile when compared with the Participatory Leader profile (OR = 2.80, *p* < 0.01) and the Low strain profile (OR = 1.93, *p* < 0.01).

Then, our results provide support for Research Question 2, showing that negative relationships at work and coworkers’ incivility distinguish profiles of the JDCS model.

### 3.4. Outcomes of Latent Profiles

Finally, we tested the strain and learning hypotheses [3] by investigating the relationships between the four extracted latent profiles and burnout (emotional exhaustion and cynicism) for the strain hypothesis and intrinsic work motivation for the learning hypothesis.

Regarding the strain hypothesis (Table 4), the Isolated Prisoner profile (M = 1.06, S.E. = 0.14) and the Moderate Strain profile (M = 0.50, S.E. = 0.04) showed the highest levels of emotional exhaustion. Both Participatory leader (M = −0.94, S.E. = 0.27) and low strain profiles (M = −0.32, S.E. = 0.05) exhibited the lowest levels of emotional exhaustion.

As for cynicism, we decided to use the DE3STEP procedure because it showed non-convergence of the model. This approach is more robust and more likely to converge as it allows equality between the variances of the cynicism across profiles [80]. Results showed that the Isolated Prisoner profile (M = 1.13, S.E. = 0.19) exhibited the highest levels of cynicism, followed by the Moderate Strain profile (M = 0.43, S.E. = 0.04). Both Participatory leader (M = −0.84, S.E. = 0.07) and low strain profiles (M = −0.25, S.E. = 0.03) exhibited the lowest levels of cynicism.

As for the learning hypothesis, results showed that the Isolated Prisoner profile (M = −0.95, S.E. = 0.16) exhibited the lowest levels of intrinsic work motivation, followed by the Moderate Strain profile (M = −0.39, S.E. = 0.05). Both Participatory leader (M = 0.78, S.E. = 0.14) and low strain profiles (M = 0.28, S.E. = 0.04) exhibited the highest levels of intrinsic work motivation.

Finally, concerning Research Question 3, our results showed that different profiles are associated to different levels of emotional exhaustion, cynicism, and intrinsic motivation.

## 4. Discussion

This study aimed at extending job stress theory and research through the identification of latent profiles of employees based on the simultaneous consideration of job demands, job control, and job support as postulated in the JDCS model [3]. A recent line of research on job stress using a person-centered approach has begun to identify empirical constellations of job stressors [2,22,23]. By means of LPA, our results have shown that four distinct profiles would better represent the job type configurations. More specifically, we were able to identify the Isolated prisoner profile, characterized by high job demands, low job control, and low (managerial and coworkers) support. This research is one of the first studies to find empirical support for this configuration, defined as iso(lated)-strain jobs in the JDCS model. Indeed, previous research found support for the high-strain profile [19,22,25,81,82], but only two studies [2,23] showed support for this profile in considering the interplay of all the components of the JDCS model. Furthermore, when compared to other studies, it should be noted that the empirical identification of this profile has similar variability in terms of size, with regards to the working population. Indeed, in our study, this profile represents 6.3% of the sample and it is in line with previous person-centered approach research, reporting an average profile size of 7% [2,18]. Other studies where social support was not included had previously showed different sizes. For example, Santos and colleagues [81] found different sizes in their study, from 9.1% among health care workers to 30.2% among urban workers. In their study, Dingemans and Henkens [82] found that the high-strain job profile contained 39% of their sample. Similarly, in his study across 27 European countries, Holman [19] found a wider size range, from 11.7% to 38.9% (mean = 18.9%). However, when defining constellations of job types, it should be noted that Holman [19] considered different components, such as a general combination of job demands and job resources.

As for the second profile, the Participatory leader is characterized by low job demands, high job control, and high support from both management and coworkers. This profile is consistent with the JDCS model and to the best of our knowledge, it is the first time this profile has been empirically supported. This profile is principally characterized by high levels of both job control and social support. In their theorization, Karasek and Theorell [3] did not offer a deep explanation of this configuration. The vast majority of studies considered the configuration of job types as theorized in the JCD model before social support was included in the model, as in the 1980s [8]. The first theorization of the JDC model became the most investigated one and it was the same for studies interested in analyzing job types as postulated in this model. Participatory leader profile is probably the less popular configuration, therefore not so many studies have been dedicated to this configuration yet.

The third profile, labelled as Moderate strain, has shown a similar shape to the isolated strain profile but with different levels of the considered variables. In fact, it was characterized by slightly above average levels of job demands and slightly below average levels of job control, and managerial and coworker support. This profile contained 37.2% of the sample and highlighted the likelihood that nuances may be found in the JDCS profiles. Furthermore, the empirical evidence of this profile emphasized the risk in dichotomizing variables and relying on cutoff values to create subgroups as postulated in the JDCS model. In fact, relying on these (too trusted) techniques forces workers into job profiles that are not universal as recently suggested [2] and empirically supported.

Finally, the last extracted profile, labelled as Low strain, was characterized by slightly below average levels of job demands, slightly above levels of job control, and moderate managerial and coworkers’ support. Our results are consistent with the JDCS model and with previous studies that adopted the person-centered approach [2,15,22,23,25,81,82]. With relation to this profile, we agree with Mauno and colleagues [2], who suggested that a low-strain profile may be a common profile. As for profile size, in our study, this constellation described 45.2% of the sample and it is therefore consistent with Santos and colleagues’ study [81], who found different sizes in their study, ranging from 39.1% among urban workers to 52.5% among teachers. As for the healthcare working population, they found that 35.2% of their sample had low-strain jobs [81].

In general, we did not identify the passive jobs, active jobs, obedient comrade, and cowboy hero constellations [3]. However, other authors adopting a person-centered approach have also failed to identify these constellations [2,15]. In this sense, our findings imply that these job types as postulated in the JDCS model are not common constellations.

Furthermore, in our study, we examined the role of interpersonal stress, such as negative relationships at work and coworker’s incivility, as predictors of these profiles. Very little research has been carried out in order to investigate predictors that would contribute to the development of these profiles. According to previous variable-centered studies [28,29,34,35,36,37,38,39], our findings have confirmed that interpersonal stressors at work would have the potential of being predictive over other job stressors [83,84,85]. In this regard, our results showed that working environments characterized by negative relationships and coworker’s incivility would increase the likelihood of Isolated prisoner and moderate strain profiles when compared to the Participatory leader and low strain profiles. Thus, our findings suggest that negative social interaction may shape the social environment where JDCS profiles develop, requiring employees to invest energy to deal with negative workplace interactions. By means of a factor mixture model, Keller and colleagues [18] showed that social stressors at work, defined as difficult interactions with supervisors and coworkers, played an important role in differentiating profiles.

Lastly, we tested both strain and learning hypotheses by taking the relationship of the extracted profiles with burnout and intrinsic work motivation, respectively, into account. More generally, Karasek and colleagues postulated that the combination of job demands and job control has a significant impact on wellbeing/health and learning outcomes [3,7,9,26]. More specifically, in the JDCS model, the strain hypothesis postulates that working in iso-strain jobs would expose workers to impaired wellbeing [3,8]. In line with the JDCS model, we found support for the strain hypothesis as both Isolated prisoner and Moderate strain profiles reported the highest levels of both emotional exhaustion and cynicism. Our results are consistent with recent person-centered approach studies [2,83]. Moreover, they confirmed that these profiles show the highest levels of emotional exhaustion when empirically identified. A large body of research has tested the iso-strain hypothesis by adopting the classical variable centered approach [12] and considering emotional exhaustion as a health outcome. Few studies considered cynicism as an outcome and to the best of our knowledge, our study is one of the first studies that aimed to investigate cynicism concerning JDCS profiles. In this regard, our results would be significant as researchers still rely on the JCD model while neglecting the role of support in shaping the constellations of job types as postulated by Karasek and colleagues [3]. Indeed, as we empirically found both the Isolated Prisoner and Moderate strain profiles, the strain hypothesis showed that as the profile configuration worsened (highest job demands, lowest job control, and lowest job support), emotional exhaustion and cynicism levels significantly increased.

Finally, the learning hypothesis proposes that jobs characterized by high job demands in combination with high job control, such as Active jobs, would lead to work motivation. Other person-centered studies were able to find this constellation [15,22], but in line with Keller and colleagues [18] and Mauno and colleagues [2], we have not found empirical support for the active profile. Furthermore, our findings highlighted the risk of using predefined cut-off points in defining this constellation. Moreover, “proposed constellations do not necessarily reflect employees’ experiences at work” [22] (p. 9). However, as no explicit hypothesis was proposed for the JDCS model and its eight constellations, we considered our study as explorative in its nature. Indeed, we found that the Participatory leader profile (lowest job demands, highest job control and social support) showed the highest levels of intrinsic work motivation. Since this profile has never been empirically identified before, it should be considered with caution. Our results are indeed not consistent with Karasek and colleagues’ hypothesis, saying that job demands are fundamental in promoting work motivation since the Participatory leader profile showed the lowest levels of job demands. In our study, the profiles characterized by high/slightly above average levels of job demands showed the lowest levels of intrinsic work motivation.

The major theoretical contribution of the present study aims at strengthening the consideration of job support as a relevant feature for job types identification, as postulated in the JDCS model. Indeed, we were able to identify the Isolated Prisoner and Participatory Leader profiles that were never found in previous studies. Based on our results, researchers should (eventually) not use the common procedure of classifying workers according to the JDC model, where only high strain, low strain, and passive and active jobs are considered. Indeed, we were not able to identify two profiles: active and passive jobs. This point is fundamental because researchers are better to consider empirically identified profiles rather than theoretically defined subgroups.

### Limitations, Future Directions, and Practical Implications

Despite the important strengths of the present study, future research should also focus on some limitations. Firstly, we considered a convenience sample of Italian healthcare workers. Further studies are required in order to investigate whether our findings would replicate using a different sample of working population and in another cultural context. Secondly, the cross-sectional nature of the data does not allow one to draw some conclusions about the directionality of the associations between the observed profiles and the outcome variables. Hence, it remains to be determined whether profiles will emerge over time. Still, future research is needed to clarify this issue and particularly, to investigate possible reciprocal relations among profiles and outcomes. Thirdly, the inclusion of potential antecedent and outcome variables was limited. In fact, by taking the variable-centered approach into account, many other antecedents (such as leadership and role stress) and outcomes (such as job satisfaction and organizational commitment) would need further investigation in future research.

In general, our findings confirm that job profiles as postulated in the JDCS model are true and that these profiles have implications in terms of workers’ health and learning outcomes. However, although empirical identification of frequent core profiles emerging in the available literature is not possible yet, the profiles that were identified in our study are partially consistent with the JDCS model. As for practical implications, organizations should consider the real nature and diffusion of the strain profiles in their workplace by adopting a person-centered approach, rather than defining them in terms of median/mean split methods. In this case, identification of empirical profiles may provide a more complete and integrated description of their organizational reality and then may help managers to implement more focused interventions.

We found that interpersonal negative relationships at work are potentially significant antecedents of profile membership. In this sense, stressful working environments, characterized by negative relationship and incivility, may have an impact on the way workers perceive job demands, job control, and social support by increasing the risk for them to be in an isolated high-strain job. Results from this study indicate that organizations and managers should consider promoting and fostering a culture of respect and social support in the workplace. Indeed, according to the “boiler room” environment metaphor [86] and the spiraling effect of incivility in the workplace [31], these high-strain environments may facilitate the development of a culture of violence and lead to a spiral of violence in the workplace. For example, organizations can implement zero tolerance for violence policy and specific human resource management practices in order to promote social support at work. Both actions are part of many healthy workplace’s models [87] aimed at building psychologically healthy workplaces. For example, in their study, Day and Nielsen [88] proposed four levels of intervention aimed at promoting healthy workplaces. Two of those kinds of interventions can be carried out at group and organizational levels: the first one would aim at fostering positive interpersonal relationships at work, while the second one would aim at building a culture of support, respect, and fairness.

Lastly, our study has shown that high job demands, low job control, and low social support clearly contributed to defining high risk profiles as leading to higher burnout and poorer intrinsic work motivation. It should then be noted that even though these patterns highlighted the complex interplay of all the JDCS components, it is still not clear whether interventions should be aimed at reducing job demands and/or increasing job control and social support. Previous researchers adopting a variable-centered approach suggested that interventions should be aimed at increasing job control as this contributes to a reduction in the perception of job demands. These interventions were consistent with the buffer hypothesis of the JDCS model, which suggested that control (and social support) can moderate the potential negative effects of high job demands on workers’ wellbeing. Recently, Mauno and colleagues [2] in their person-centered study suggested that “stress interventions intended to improve job control may be particularly beneficial: job control is changeable, and improvements therein are likely to lead to positive employee outcomes” (p. 13).

## Figures and Tables

**Table 1:** Means (M), Standard Deviations (SD), and correlations between study variables.

	M	SD	1	2	3	4	5	6	7	8	9
1. Job demands	2.77	0.81	(0.88)								
2. Job Control	3.22	0.81	−0.35	(0.82)							
3. Managerial support	3.30	0.94	−0.29	0.40	(0.84)						
4. Coworkers’ support	3.73	0.82	−0.23	0.34	0.57	(0.82)					
5. Negative Relatationships at work	2.30	0.80	0.36	−0.32	−0.42	−0.49	(0.76)				
6. Coworkers’ incivility	1.02	1.15	0.21	−0.26	−0.32	−0.51	0.51	(0.89)			
7. Intrinsic work motivation	4.79	1.29	−0.13	0.27	0.33	0.29	−0.21	−0.18	(0.91)		
8. Emotional exhaustion	2.25	1.30	0.57	−0.32	−0.33	−0.28	0.40	0.30	−0.36	(0.78)	
9. Cynicism	0.99	1.13	0.45	−0.28	−0.27	−0.24	0.34	0.30	−0.32	0.61	(0.73)

Note: N = 1671. McDonald’s ω coefficients are reported in italics along the diagonal.

**Table 2:** Fit indices, entropy, and model comparisons for estimated latent profile analyses models.

Model	LL	#fp	Scaling	AIC	CAIC	BIC	ABIC	Entropy	aLMR	BLRT
1 Profile	−8957.20	8	0.993	17,930.41	17,981.78	17,973.78	17,948.36	Na	Na	Na
2 Profiles	−8495.47	13	1.185	17,016.94	17,100.42	17,087.42	17,046.12	0.63	<0.001	<0.001
3 Profiles	−8343.79	18	1.426	16,723.57	16,839.15	16,821.15	16,763.97	0.71	<0.001	<0.001
4 Profiles	−8287.43	23	1.307	16,620.86	16,768.55	16,745.55	16,672.48	0.70	<0.01	<0.001
5 Profiles	−8249.71	28	1.269	16,555.43	16,735.22	16,707.22	16,618.27	0.73	<0.05	<0.001
6 Profiles	−8223.16	33	1.177	16,512.32	16,724.21	16,691.21	16,586.38	0.76	>0.05	<0.001
7 Profiles	−8195.42	38	1.210	16,466.84	16,710.85	16,672.85	16,552.13	0.75	>0.05	<0.001
8 Profiles	−8174.03	43	1.331	16,434.05	16,710.16	16,667.16	16,530.56	0.73	>0.05	<0.001

Note: LL = Log-likelihood; #fp = number of free parameters; AIC = Akaike Information Criterion; CAIC = Constant AIC; BIC = Bayesian Information Criterion; ABIC = Adjusted BIC; aLMRT = Adjusted Vuong-LoMendell-Rubin test; BLRT = Bootstrap Likelihood Ratio Test.

**Table 3:** The three-step procedure results for antecedents (R3STEP).

	Profile 1 vs. 2		Profile 1 vs. 3		Profile 1 vs. 4		Profile 2 vs. 4		Profile 3 vs. 2		Profile 3 vs. 4	
	Estimate (S.E.)	OR	Estimate (S.E.)	OR	Estimate (S.E.)	OR	Estimate (S.E.)	OR	Estimate (S.E.)	OR	Estimate (S.E.)	OR
Negative relationships at work	4.60 (0.63)	99.48 *	1.62 (0.34)	5.05 *	3.31 (0.41)	27.47 *	1.29 (0.47)	3.63 *	2.98 (0.53)	19.65 *	1.69 (0.22)	5.42 *
Coworker incivility	1.65 (0.46)	5.18 *	0.62 (0.10)	1.85 *	1.27 (0.26)	3.56 *	0.38 (0.41)	1.46 **	1.03 (0.40)	2.80 *	0.66 (0.15)	1.93 *

Note: Positive coefficient values indicate that higher values on the antecedent make a person more likely to be in the first latent profile of the two being compared; negative values indicate that higher values on the antecedent make a person more likely to be in the second latent profile compared. Estimate = estimate (β) from the R3STEP multinomial logistic regression analysis; S.E. = Standard Error; OR = Odd Ratio; Profile 1 = Isolated Prisoner; Profile 2 = Participatory Leader; Profile 3 = Moderate Strain; Profile 4 = Low strain. * *p* < 0.01; ** *p* > 0.05.

**Table 4:** Outcome means and pairwise comparisons between profiles.

	Profile 1	Profile 2	Profile 3	Profile 4	Differences between Profiles
	Mean (S.E.)	Mean (S.E.)	Mean (S.E.)	Mean (S.E.)	
Emotional Exhaustion	1.06(0.14)	−0.94(0.27)	0.50(0.04)	−0.32(0.05)	1 > 3 > 4 > 2
Cynicism	1.13(0.19)	−0.84(0.07)	0.43(0.04)	−0.25(0.03)	1 > 3 > 4 > 2
Intrinsic motivation	−0.95(0.16)	0.78(0.14)	−0.39(0.05)	0.28(0.04)	1 < 3 < 4 < 2

Note: S.E. = Standard Error; Profile 1 = Isolated Prisoner; Profile 2 = Participatory Leader; Profile 3 = Moderate Strain; Profile 4 = Low strain.
